# Conservation Hotspots for the Turtles on the High Seas of the Atlantic Ocean

**DOI:** 10.1371/journal.pone.0133614

**Published:** 2015-08-12

**Authors:** Hsiang-Wen Huang

**Affiliations:** Institute of Marine Affairs and Resource Management, National Taiwan Ocean University, Keelung, Taiwan; Deakin University, AUSTRALIA

## Abstract

Understanding the distribution of bycaught sea turtles could inform conservation strategies and priorities. This research analyses the distribution of turtles caught as longline fisheries bycatch on the high seas of the Atlantic Ocean. This research collected 18,142 bycatch observations and 47.1 million hooks from large-scale Taiwanese longline vessels in the Atlantic Ocean from June 2002 to December 2013. The coverage rates were ranged from 0.48% to 17.54% by year. Seven hundred and sixty-seven turtles were caught, and the major species were leatherback (59.8%), olive ridley (27.1%) and loggerhead turtles (8.7%). Most olive ridley (81.7%) and loggerhead (82.1%) turtles were hooked, while the leatherbacks were both hooked (44.0%) and entangled (31.8%). Depending on the species, 21.4% to 57.7% were dead when brought onboard. Most of the turtles were caught in tropical areas, especially in the Gulf of Guinea (15°N-10°S, 30°W-10°E), but loggerheads were caught in the south Atlantic Ocean (25°S-35°S, 40°W-10°E and 30°S-40°S, 55°W-45°W). The bycatch rate was the highest at 0.030 per 1000 hooks for leatherbacks in the tropical area. The bycatch rates of olive ridley ranged from 0 to 0.010 per thousand hooks. The loggerhead bycatch rates were higher in the northern and southern Atlantic Ocean and ranged from 0.0128 to 0.0239 per thousand hooks. Due to the characteristics of the Taiwanese deep-set longline fleet, bycatch rates were lower than those of coastal longline fisheries, but mortality rates were higher because of the long hours of operation. Gear and bait modification should be considered to reduce sea turtle bycatch and increase survival rates while reducing the use of shallow hooks would also be helpful.

## Introduction

The populations of many sea turtles are decreasing due to marine pollution, tourism, coastal development, plastic bags/debris, boat collisions, fisheries bycatch, direct take of adults, egg collection by humans, egg predation by animals, nest loss due to hurricanes, etc. [1,2]. There are six species of sea turtle in the Atlantic Ocean, and all are listed on the IUCN Red List as either vulnerable (leatherback, *Dermochelys coriacea*, and olive ridley, *Lepidochelys olivacea*)[3,4], endangered (loggerhead, *Caretta caretta*, and green turtle, *Chelonia mydas*)[5,6], or critically endangered (Kemp’s ridley, *Lepidochelys kempii*, and hawksbill, *Eretmochelys imbricata*)[7,8]. To improve our knowledge of these migratory species and provide conservation recommendations, the IUCN Marine Turtle Specialist Group convened the Burning Issues Working Group (MTSG_BI), which developed Regional Management Units (RMUs) to serve as conservation units. Eleven of the RMUs for the most endangered marine turtles should be prioritized for conservation based on their high risk and threat scores, including an RMU for hawksbill in the East Atlantic Ocean and one for loggerhead in the northeast Atlantic Ocean (Cape Verde) [9].

Among the threats to sea turtles, many fishing activities, such as with trawls, gillnets, pound nets, purse seines and lines, has caused the decline of some populations [10]; thus, estimating the mortality from bycatch is important for understanding the threats to sea turtles. The reported global bycatch of turtles reached 85,000 individuals in gillnet, longline, and trawl fisheries from 1990 to 2008, but the figure is likely an underestimate due to poor coverage and the lack of information from small-scale fisheries [9,11]. The bycatch rate was highest in the northwest Atlantic (0.5954 per thousand hooks), Mediterranean (0.2740 per thousand hooks), and southwest Atlantic (0.2240 per thousand hooks), and the bycatch rates were lower in West Africa (0.0356 per thousand hooks) and the northeast Atlantic Ocean (0.0367 per thousand hooks)[11]. Among the six turtle species in the Atlantic Ocean, loggerhead and leatherback turtles were the most frequency caught [12,13].

The longline fishery is considered to have the highest bycatch impact, but this is likely the result of the greater availability of longline fisheries observer bycatch reports [14]. However, most information about longline sea turtle bycatch has come from coastal areas [11,15,16], and there has been limited information from the large-scale industrial fleets operating on the high seas. In the Atlantic Ocean, a public domain ICCAT spatial database showed the top five longline fishing countries were Taiwan, Japan, Spain, Belize, and China between 2002 to 2013 [17]. Among them, the Taiwanese fleet is one of the largest distant-water longline efforts operating in the entire Atlantic Ocean [18]. This study used observer data from 2002 to 2013 and effort data from the Taiwanese fleet to (1) understand the biological and spatio-temporal characteristics of sea turtle bycatch, (2) analyze the distribution of sea turtle bycatch by species, (3) estimate sea turtle bycatch rates from the large-scale, deep-set longline fleet, and (4) identify hotspots for strengthened management of fisheries activities.

## Materials and Methods

### Fisheries and study areas

There are three large-scale Taiwanese longline tuna fleets operating in the international waters of the Atlantic Ocean. The bigeye (*Thunnus obesus*) fishing fleet works in tropical waters between 15°N and 15°S, and the northern and southern albacore (*T*. *alalunga*) fleets operate at higher latitudes in either the northern Atlantic (north of 15°N) or the southern Atlantic (south of 15°S).

The bigeye fishing vessels studied ranged from 400–700 gross register tonnage (GRT) and 40–57 m in length, and they used a monofilament longline system with 4–4.2 inches non-offset J hooks with squid, mackerel, sardine, or mixed baits. In each set, approximately 2900 hooks on 175 km of longline were deployed on average, and the secondary branch lines, which attach to the main line, averaged approximately 54 m in length. Operations started at 0300–0600 and lasted for approximately 6.9 hours; hauling began between 1000 and 1300 and lasted for approximately 17.5 hours. The estimated fishing depths were more than 100 m.

The size of the north albacore vessels has changed over time. In the early 2000s, the vessels were approximately 300 GRT and 40 m in length, but in recent years, those vessels have been replaced by larger vessels that are 730 GRT and 48 m in length. During the study period, they used a monofilament longline system with 2’ or 3.2’ inches non-offset J hooks with sardine, milkfish, mackerel, saury, scad, and mixed baits. In each set, 3290 hooks on 150 km of longline were deployed on average, and the secondary branch lines averaged approximately 38 m. Line setting started at 2300–0300 and lasted for 6.8 hours with approximately 14.3 hours spent for hauling.

The south albacore vessels were 190–363 GRT and 33–42 m in length. They also used a monofilament longline system with 2’ or 3.2’ non-offset J hooks with sardine, milkfish, mackerel, saury, scad, and mixed baits. In each set, 3240 hooks on 130 km of longline were deployed on average, and the secondary branch lines averaged approximately 25 m. Line setting started at 0400–0600 and lasted for 6.3 hours with approximately 13.9 hours spent on hauling.

### Data Collection

Taiwan’s large-scale tuna longline vessels’ observer program started in 2002. The observer deployment was designed to be sampling stratified by fleet. The coverage rates were low in the beginning due to the budget limitations, and increased to 5% in accordance with recommendations adopted by ICCAT. In addition, the bigeye tuna fleet was requested for 100% observer coverage rate in 2006 and 10% in 2007[19–21]. The target observer coverage rates for albacore fleets are 5% and 10% for bigeye fleets after 2006.

Sea turtle bycatch data were collected by onboard observers who recorded the location of fishing operations (latitude and longitude at the start and end of setting and hauling), number of hooks deployed, time of setting and hauling, sea surface temperature (SST), bait types, catch information (species, number, status, length, weight, and gender), and bycatch information (number, species, status (dead/alive), and gender, if possible, for seabirds, sea turtles, and cetaceans). The curved carapace lengths (CCL) of turtles brought on board were measured, and when possible, entangled hooks and lines were removed prior to release. The manner of bycatch (hooked, entangled, etc.) was recorded after 2010. Turtles not brought on board, for which species, status, and gender could not be identified, were recorded as “unknown”.

To estimate the observer coverage, fishing effort data were collected from vessel logbooks, which record the position of sets (latitude and longitude), number of hooks, number and weight of the catch by species, and the lengths of the first 30 fish. Some vessels had monitoring systems that automatically reported their position, and those systems were used to verify the logbook records. The Fisheries Agency of Taiwan requests submission of logbook data, which are processed by the Overseas Fisheries Development Council of the Republic of China. The effort data will be extrapolated to the whole fleet if not 100% covered and summarized as hooks per a 5 by 5 degree square grid by month in accordance with the data requirements of the International Convention for the Conservation of Atlantic Tuna (ICCAT). These effort, catch, and length data summaries are submitted annually to ICCAT and can be downloaded from the ICCAT website (http://www.iccat.int/en/accesingdb.htm).

In addition, to understand the overlap of sea turtle bycatch with turtle distributions and RMUs [22], the turtle distribution and RMU data files were collected from SWOT/OBIS_SEAMAP on 12 September, 2014 [23, 24].

### Data Analysis

#### Spatial-temporal distribution

**Fig 1:**
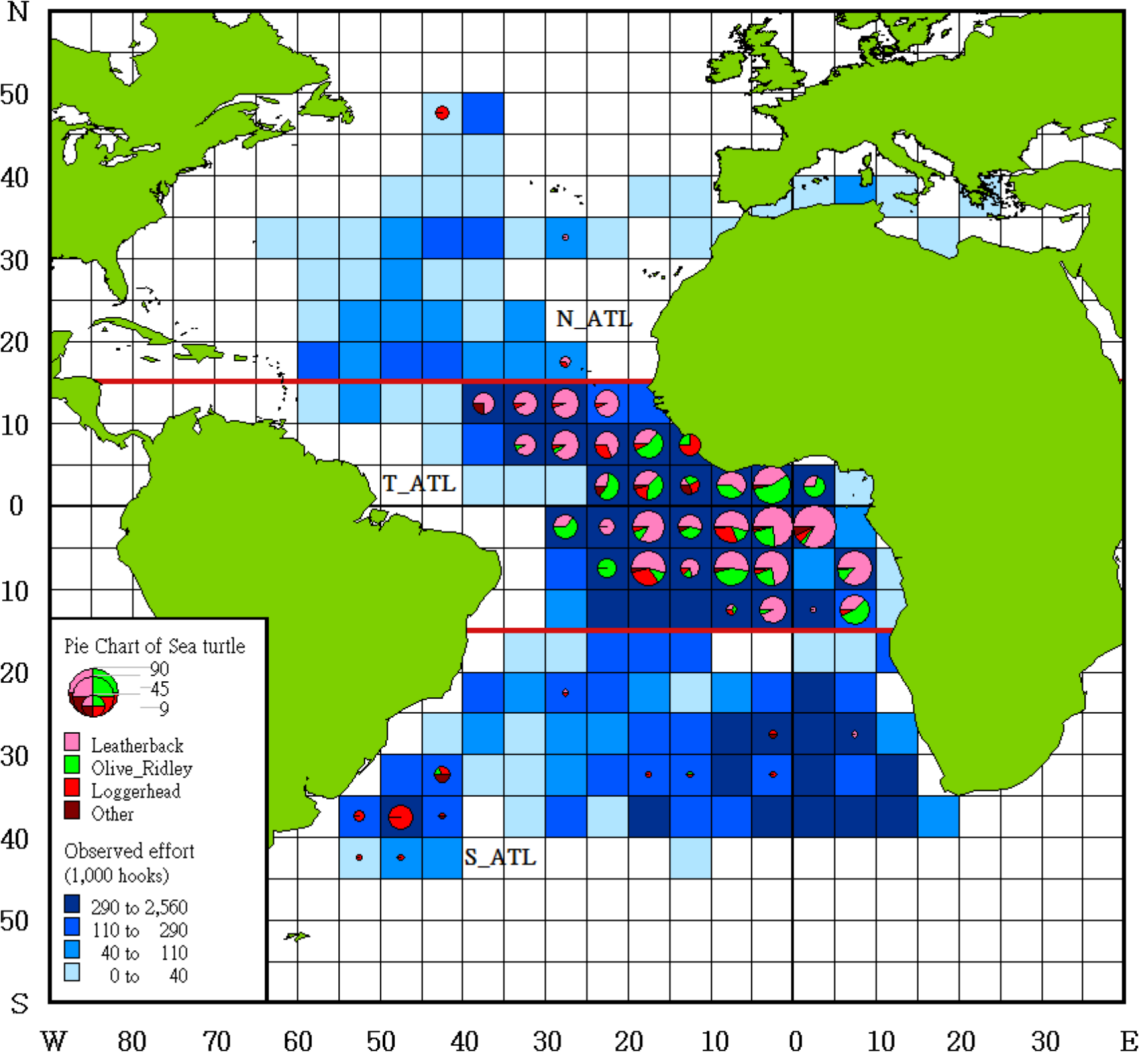
Observed effort and number of sea turtle bycatch distribution by species in 5*5-degree grid from 2002 to 2013 in all Taiwanese fleets.

Given that previous studies have revealed spatial-temporal differences in the distribution of sea turtle RMUs and bycatch rates [9,15], the data were stratified. For temporal factors, we separated the years into 4 quarters: 1st quarter (January~ March), 2nd quarter (April~ June), 3rd quarter (July~ September), and 4th quarter (October~ December). Spatial data were stratified into three areas: the north Atlantic Ocean (**N_ATL**, north of 15° N), the tropical Atlantic Ocean (**T_ATL**, between 0–15° N and 0–15° S, and the south Atlantic Ocean (**S_ATL**, south of 15° S) (Fig 1).

The locations (latitude and longitude) of sea turtles bycatch and effort (hooks summarized by 5*5 degree) were mapped to show the seasonal distribution by MAPINFO Version 10. The distribution of turtle RMUs are overlapped to understand the encounter situation for different RMUs.

### Estimation of bycatch rate

The rate of turtle bycatch (bycatch per unit effort, BPUE) *r* was calculated as the number of sea turtles caught per 1,000 hooks for each species per area per year [25–29].

$$\overset{⌢}{r}=\frac{\sum_{i}t_{i}}{\sum_{i}h_{i}}$$

where t_*i*_ and *h*_*i*_ are the number of bycatch turtles and the hooks observed for *i* set within one year one area. Observed sets and coverage rates are important factors for estimate the bycatch rates. At least 30 sets of observations are required within a unit (year-area-quarter) for [30, 31]. If there is insufficiency observation for a stratum, the data will be pooled. The binomial estimator with Clopper-Pearson confidence intervals was used to determine the variation in bycatch sea turtle mortality in each stratum using the R program [28, 29, 32].

To determine the significance of impact for year, quarter, area, and SST on bycatch rates, multivariate analysis of variance (MANOVA) is applied [33]. Four independent variables included year (2002–2013), quarter (1–4), area (N_ATL, T_ATL, and S_ATL), and SST (13–36°C). The dependent variable is bycatch rates of three major turtle species. Because the bycatch rate did not fall under a normal distribution, it is log-transformed to fit the model and a constant (10% of mean bycatch rate) was added to avoid the zero value. Statistical analyses were performed using the SAS version 9.4 (SAS Institute, Cary, NC).

## Results

### Distribution of observed fishing effort

The total number of fishing vessels was 170 in 2002, and it decreased to 124 in 2012 [34,35]. Fishing effort of all fleets decreased from 136.65 million hooks in 2002 to 50.12 million hooks in 2013(Table 1). T_ATL was the primary fishing ground and accounted for 58.2% of the total effort followed by S_ATL (34.3%) and N_ATL (7.4%).

**Table 1:** Observed number of sets, efforts, total number of efforts and observer coverage rates for the Taiwanese longline fleet in the Atlantic Ocean from 2002 to 2013.

Year	Observed sets	Observed efforts (1000 hooks)	Total efforts (million hooks)	Observer coverage by hooks (%)				
				Jan-Mar	Apr-Jun	Jul- Sep	Oct-Dec	Whole
**2002**	**206**	**641.8**	**136.65**	**0.00**	**0.18**	**1.22**	**1.01**	**0.48**
**2003**	**255**	**900.7**	**160.64**	**0.00**	**0.84**	**1.26**	**0.27**	**0.56**
**2004**	**622**	**2274.5**	**121.05**	**0.12**	**2.22**	**3.46**	**2.24**	**1.88**
**2005**	**395**	**1370.7**	**89.03**	**0.00**	**0.35**	**1.45**	**5.15**	**1.54**
**2006**	**3263**	**10376.9**	**59.15**	**21.68**	**16.60**	**13.71**	**20.43**	**17.54**
**2007**	**1798**	**5860.9**	**67.02**	**9.29**	**5.56**	**10.43**	**9.86**	**8.74**
**2008**	**1475**	**4069.2**	**54.72**	**2.93**	**8.05**	**6.94**	**16.00**	**7.44**
**2009**	**1553**	**4018.4**	**62.74**	**9.15**	**4.11**	**5.83**	**6.69**	**6.40**
**2010**	**1890**	**4608.7**	**68.64**	**5.54**	**8.36**	**6.82**	**6.13**	**6.72**
**2011**	**2571**	**6291.5**	**82.88**	**3.19**	**3.67**	**10.72**	**13.69**	**7.60**
**2012**	**2324**	**5205.4**	**64.51**	**9.60**	**8.38**	**8.23**	**4.87**	**9.96**
**2013** *	**1791**	**1472.2**	**50.12**	**0.46**	**3.50**	**5.98**	**2.13**	**2.93**
**Total**	**18142**	**47088.3**	**1017.18**	**3.42**	**4.23**	**5.53**	**5.84**	**4.63**

* The coverage rate of 2013 was preliminary because the observed and total efforts were both preliminary.

Taiwanese onboard observers recorded 18,142 sets and 47.1 million hooks from June 2002 to December 2013 (Table 1). The observed coverage rate by hooks was 4.63% across all years and increased from 0.48% in 2002 to 9.96% in 2012. The coverage rate in 2013 was preliminary because not all of the observer and effort data were recovered. The distribution of the observed effort is shown in Fig 1. Most of the effort was in T_ATL (68.8%) followed by S_ATL (26.5%) and N_ATL (4.7%). The quarterly observer coverage ranged from 3.42% in first quarter to 5.84% in fourth quarter.

Of the total observed sets, only 587 (3.8%) recorded bycatch of turtles, and during these sets, a total of 767 sea turtles were caught. The number of incidentally caught turtles ranged from 0 to 7 per set, and most (710, 92.5%) were distributed in the T_ATL (Fig 1).

The observer coverage rates, bycatch rates and 95% confidence interval per area, year, and species were listed in Table 2. The observer coverage rates ranged from 3.4–16.4% in N_ATL, 0.2%-68.3% in T_ATL, and 0.6%-8.4% in S_ATL (Table 2). The number of observed hooks was highest in T_ATL and the coverage rate was highest in N_ATL.

**Table 2:** Number of observed hooks, captures and BPUE (bycatch per unit effort) per area and quarter of the Taiwanese longline fleet in the Atlantic Ocean between 2002 and 2013.

Area	Year	Observed thousand hooks	Coverage rate	Leatherback			Olive ridley			Loggerhead		
				Capture	BPUE	95% CI	Capture	BPUE	95% CI	Capture	BPUE	95% CI
**N_ATL (north of 15° N)**	**2004**	**390.8**	**3.5%**	**2**	**0.0051**	**0.0006–0.0185**	**0**	**0.0000**		**5**	**0.0128**	**0.0042–0.0299**
	**2006**	**192.3**	**3.4%**	**2**	**0.0104**	**0.0013–0.0376**	**0**	**0.0000**		**0**	**0.0000**	
	**2007**	**421.4**	**12.3%**	**0**	**0.0000**		**0**	**0.0000**		**0**	**0.0000**	
	**2008**	**91.6**	**4.2%**	**0**	**0.0000**		**0**	**0.0000**		**0**	**0.0000**	
	**2009**	**298.3**	**13.8%**	**0**	**0.0000**		**0**	**0.0000**		**1**	**0.0034**	**0.0000–0.0187**
	**2010**	**419.1**	**16.4%**	**0**	**0.0000**		**0**	**0.0000**		**0**	**0.0000**	
	**2012**	**307.4**	**14.2%**	**0**	**0.0000**		**0**	**0.0000**		**0**	**0.0000**	
	**2013**	**111.9**	**4.1%**	**0**	**0.0000**		**0**	**0.0000**		**0**	**0.0000**	
**T_ATL (between 15° N and 15° S)**	**2002**	**332.9**	**0.5%**	**9**	**0.0270**	**0.0124–0.0513**	**1**	**0.0030**	**0.0000–0.0167**	**1**	**0.0030**	**0.0000–0.0167**
	**2003**	**233.3**	**0.2%**	**7**	**0.0300**	**0.0120–0.0618**	**0**	**0.0000**		**0**	**0.0000**	
	**2004**	**658.5**	**0.8%**	**4**	**0.0061**	**0.0016–0.0156**	**1**	**0.0015**	**0.0000–0.0085**	**1**	**0.0015**	**0.0000–0.0085**
	**2005**	**1108.6**	**2.1%**	**26**	**0.0235**	**0.0153–0.0343**	**3**	**0.0027**	**0.0006–0.0079**	**1**	**0.0009**	**0.0000–0.0050**
	**2006**	**9598.9**	**68.3%**	**218**	**0.0227**	**0.0198–0.0259**	**40**	**0.0042**	**0.0030–0.0057**	**12**	**0.0013**	**0.0006–0.0022**
	**2007**	**3912.3**	**10.4%**	**16**	**0.0041**	**0.0023–0.0066**	**21**	**0.0054**	**0.0033–0.0082**	**1**	**0.0003**	**0.0000–0.0014**
	**2008**	**2584.2**	**8.2%**	**57**	**0.0221**	**0.0167–0.0286**	**60**	**0.0232**	**0.0178–0.0299**	**1**	**0.0004**	**0.0000–0.0022**
	**2009**	**2617.7**	**6.1%**	**10**	**0.0038**	**0.0018–0.0070**	**25**	**0.0096**	**0.0062–0.0141**	**0**	**0.0000**	
	**2010**	**3296.5**	**6.9%**	**24**	**0.0073**	**0.0047–0.0108**	**3**	**0.0009**	**0.0002–0.0027**	**9**	**0.0027**	**0.0012–0.0052**
	**2011**	**4280.7**	**7.7%**	**15**	**0.0035**	**0.0020–0.0058**	**16**	**0.0037**	**0.0021–0.0061**	**1**	**0.0002**	**0.0000–0.0013**
	**2012**	**3146.5**	**7.6%**	**64**	**0.0203**	**0.0157–0.0260**	**32**	**0.0102**	**0.0070–0.0144**	**1**	**0.0003**	**0.0000–0.0018**
	**2013**	**604.1**	**2.1%**	**1**	**0.0017**	**0.0000–0.0092**	**3**	**0.0050**	**0.0010–0.0145**	**0**	**0.0000**	
**S_ATL (south of 15° S)**	**2002**	**308.9**	**0.6%**	**0**	**0.0000**		**1**	**0.0032**	**0.0000–0.0180**	**3**	**0.0097**	**0.0020–0.0284**
	**2003**	**667.4**	**1.3%**	**2**	**0.0030**	**0.0003–0.0108**	**0**	**0.0000**		**0**	**0.0000**	
	**2004**	**1225.3**	**4.4%**	**0**	**0.0000**		**0**	**0.0000**		**0**	**0.0000**	
	**2005**	**262.1**	**0.9%**	**1**	**0.0038**	**0.0000–0.0213**	**0**	**0.0000**		**0**	**0.0000**	
	**2006**	**585.6**	**1.5%**	**0**	**0.0000**		**0**	**0.0000**		**14**	**0.0239**	**0.0131–0.040**
	**2007**	**1527.3**	**5.9%**	**0**	**0.0000**		**0**	**0.0000**		**0**	**0.0000**	
	**2008**	**1393.4**	**6.7%**	**0**	**0.0000**		**0**	**0.0000**		**1**	**0.0007**	**0.0000–0.0040**
	**2009**	**1099.6**	**6.3%**	**0**	**0.0000**		**0**	**0.0000**		**0**	**0.0000**	
	**2010**	**893.0**	**4.9%**	**1**	**0.0011**	**0.0000–0.0062**	**1**	**0.0011**	**0.0000–0.0018**	**11**	**0.0123**	**0.0061–0.0220**
	**2011**	**2010.8**	**7.9%**	**0**	**0.0000**		**0**	**0.0000**		**0**	**0.0000**	
	**2012**	**1751.6**	**8.4%**	**0**	**0.0000**		**1**	**0.0006**	**0.0000–0.0032**	**0**	**0.0000**	
	**2013**	**756.2**	**4.2%**	**0**	**0.0000**		**0**	**0.0000**		**4**	**0.0053**	**0.0014–0.0135**

The ANOVA results of the multivariate variance analysis of leatherback and olive ridley indicate the significant difference between years, quarters and areas (Table A and Table B in S1 Appendix). For loggerheads, there is no significant difference between areas and quarters, but there is a significant difference between years and SST (Table C in S1 Appendix).

The bycatch rate was highest at 0.030 per 1000 hooks for leatherback in the tropical area. The bycatch rates of olive ridley were low in N_ATL and S_ATL, and ranged from 0 to 0.0232 per thousand hooks in tropical Atlantic Ocean. The loggerhead bycatch rates were higher in N_ATL and S_ATL which was around 0.0128 to 0.0239 per thousand hooks.

### Characteristics of incidentally caught turtles by species

Leatherbacks (n = 459, 59.8%) were the most common turtle species captured followed by olive ridleys (n = 208, 27.1%), loggerheads (n = 67, 8.7%), green turtles (n = 14, 1.8%), hawksbills (n = 6, 0.8%), and unidentified species (n = 13, 1.7%). The bycatch number, onboard status, bycatch reason, and gender by species are listed in Tables 3–5.

**Table 3:** Number and percentage of bycatch turtles by onboard status for each species in the Atlantic Ocean from 2002 to 2013. The high percentage of unknown data points is due, in part, to the fact that many turtles were discarded before being brought onboard due to larger size and other reasons.

Species	Alive	(%)	Dead	(%)	Unknown	(%)	Sum
**Leatherback**	**240**	**52.3%**	**98**	**21.4%**	**121**	**26.4%**	**459**
**Olive Ridley**	**47**	**22.6%**	**120**	**57.7%**	**41**	**19.7%**	**208**
**Loggerhead**	**37**	**55.2%**	**23**	**34.3%**	**7**	**10.4%**	**67**
**Green Turtle**	**7**	**50.0%**	**5**	**35.7%**	**2**	**14.3%**	**14**
**Hawksbill**	**6**	**100.0%**					**6**
**Unknown**	**6**	**46.2%**	**2**	**15.4%**	**5**	**38.5%**	**13**

**Table 4:** Number and percentage of bycatch reason for each species in the Atlantic Ocean from 2002 to 2013. The high percentage of unknown data points is due, in part, to the fact that the manner of bycatch was not recorded before 2010, and many turtles were discarded before being brought onboard due to larger size and other reasons.

Species	hooked	(%)	Entangle	(%)	Unknown	(%)	Sum
**Leatherback**	**202**	**44.0%**	**146**	**31.8%**	**111**	**24.2%**	**459**
**Olive ridley**	**170**	**81.7%**	**7**	**3.4%**	**31**	**14.9%**	**208**
**Loggerhead**	**55**	**82.1%**	**5**	**7.5%**	**7**	**10.4%**	**67**
**Green turtle**	**11**	**78.6%**	**1**	**7.1%**	**2**	**14.3%**	**14**
**Hawksbill**	**4**	**66.7%**	**2**	**33.3%**			**6**
**Unknown**	**5**	**38.5%**	**4**	**30.8%**	**4**	**30.8%**	**13**

**Table 5:** Number and percentage of bycatch gender for each species in the Atlantic Ocean from 2002 to 2013. The high percentage of unknown data points is due, in part, to the fact that many turtles were discarded before being brought onboard due to larger size and other reasons.

Species	Female	(%)	Male	(%)	Unknown	(%)	Sum
**Leatherback**	**39**	**8.5%**	**35**	**7.6%**	**385**	**83.9%**	**459**
**Olive Ridley**	**57**	**27.4%**	**35**	**16.8%**	**116**	**55.8%**	**208**
**Loggerhead**	**13**	**19.4%**	**6**	**9.0%**	**48**	**71.6%**	**67**
**Green Turtle**	**3**	**21.4%**	**1**	**7.1%**	**10**	**71.4%**	**14**
**Hawksbill**					**6**	**100.0%**	**6**
**Unknown**	**1**	**7.7%**	**3**	**23.1%**	**9**	**69.2%**	**13**

#### Leatherback bycatch

**Fig 2:**
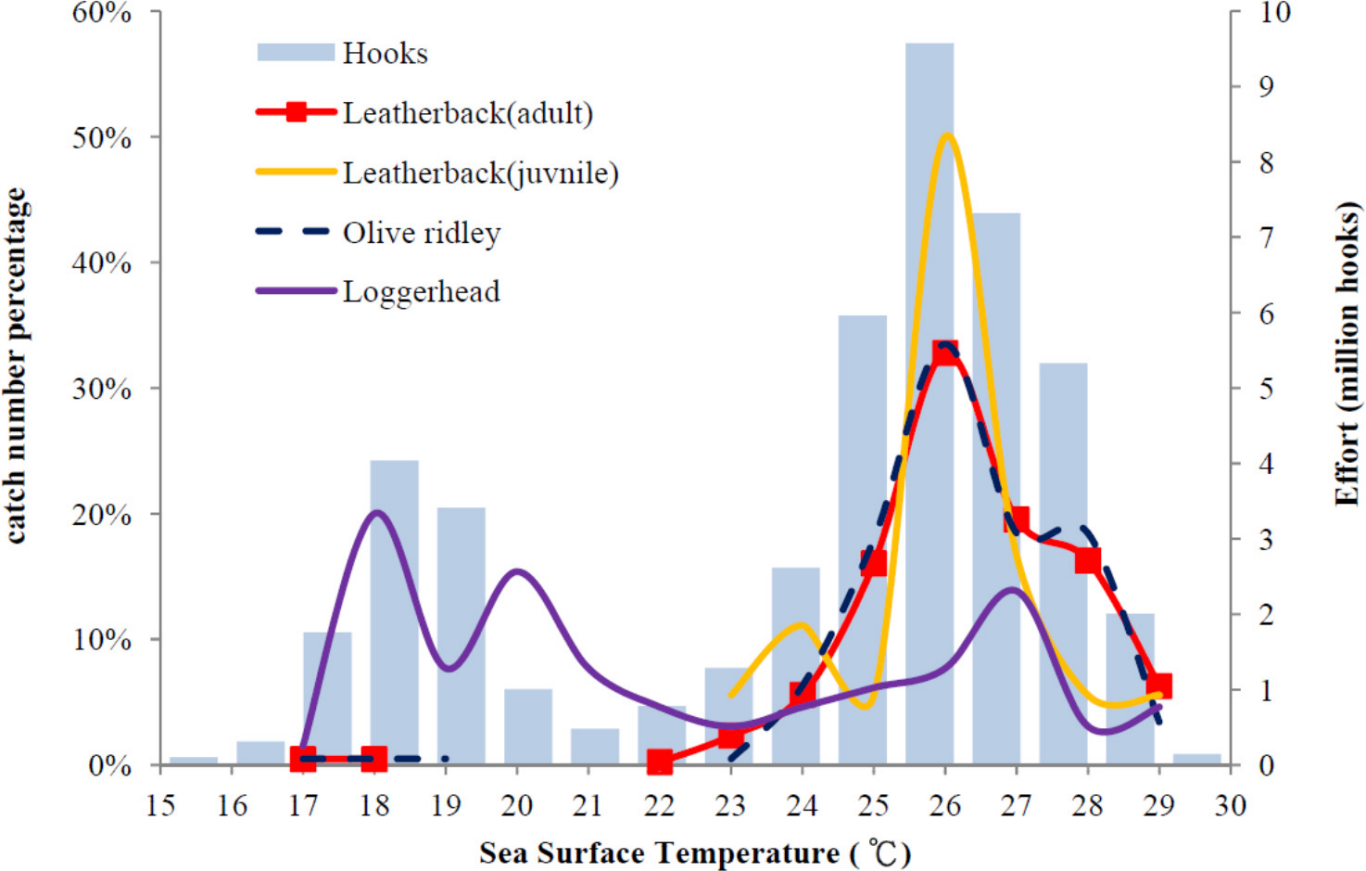
Catch percentage for three major bycatch turtle species and total observed effort relative to sea surface temperature.

**Fig 3:**
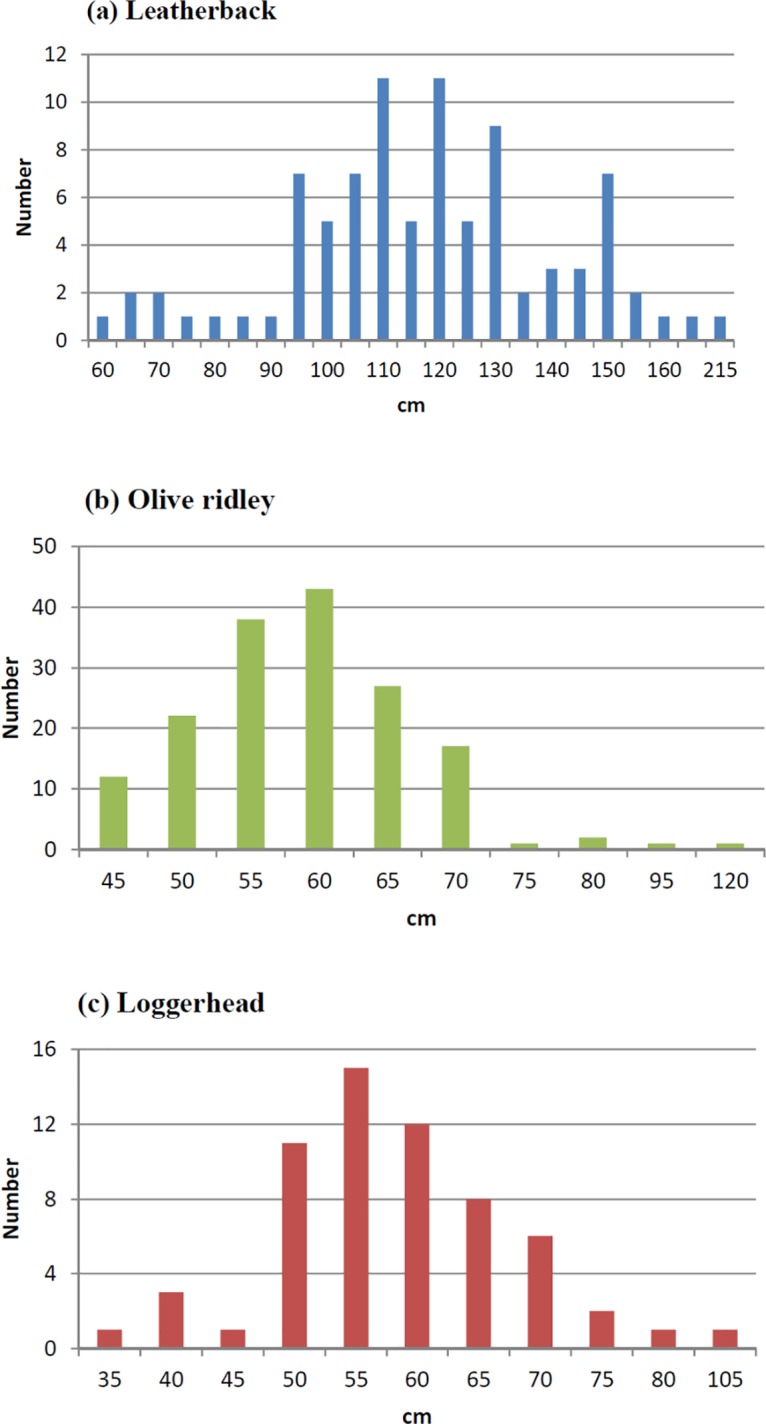
Curved carapace length (CCL) of three major sea turtles caught in Taiwanese longline fishery, (A) Leatherback; (B) Olive ridley; (C) Loggerhead. These are the distributions for those measured onboard. Some larger leatherbacks could not be measured because they were released before being placed onboard.

Leatherbacks were caught in waters ranging in temperature from 16.9–29.2°C with the majority being found in 26°C waters. Previous research showed smaller leatherbacks only distributed in warmer water [36]. We separated the leatherbacks into juveniles (CCL<100 cm), adults (CCL>100cm and those too big to fit onboard) (Fig 2). Around 80% juveniles were caught in water warmer than 26°C. Only 22% were distributed in 23–25°C waters. Fewer adult leatherbacks were caught in 17°C. The mortality rate was 21.4% (Table 3), and the majority of leatherbacks (44.0%) were hooked while 31.8% were entangled, which was higher than for most of the other species (Table 4). Among the 16.1% whose genders were identified, 8.5% were female, and 7.6% were male (Table 5). Among the 94 whose CCLs were measured, the average length was 118.58 cm (62–216 cm range, SD: 25.64 cm, Fig 3A). However, the average length may be an underestimate because many of the larger leatherbacks were released without coming on board.

**Fig 4:**
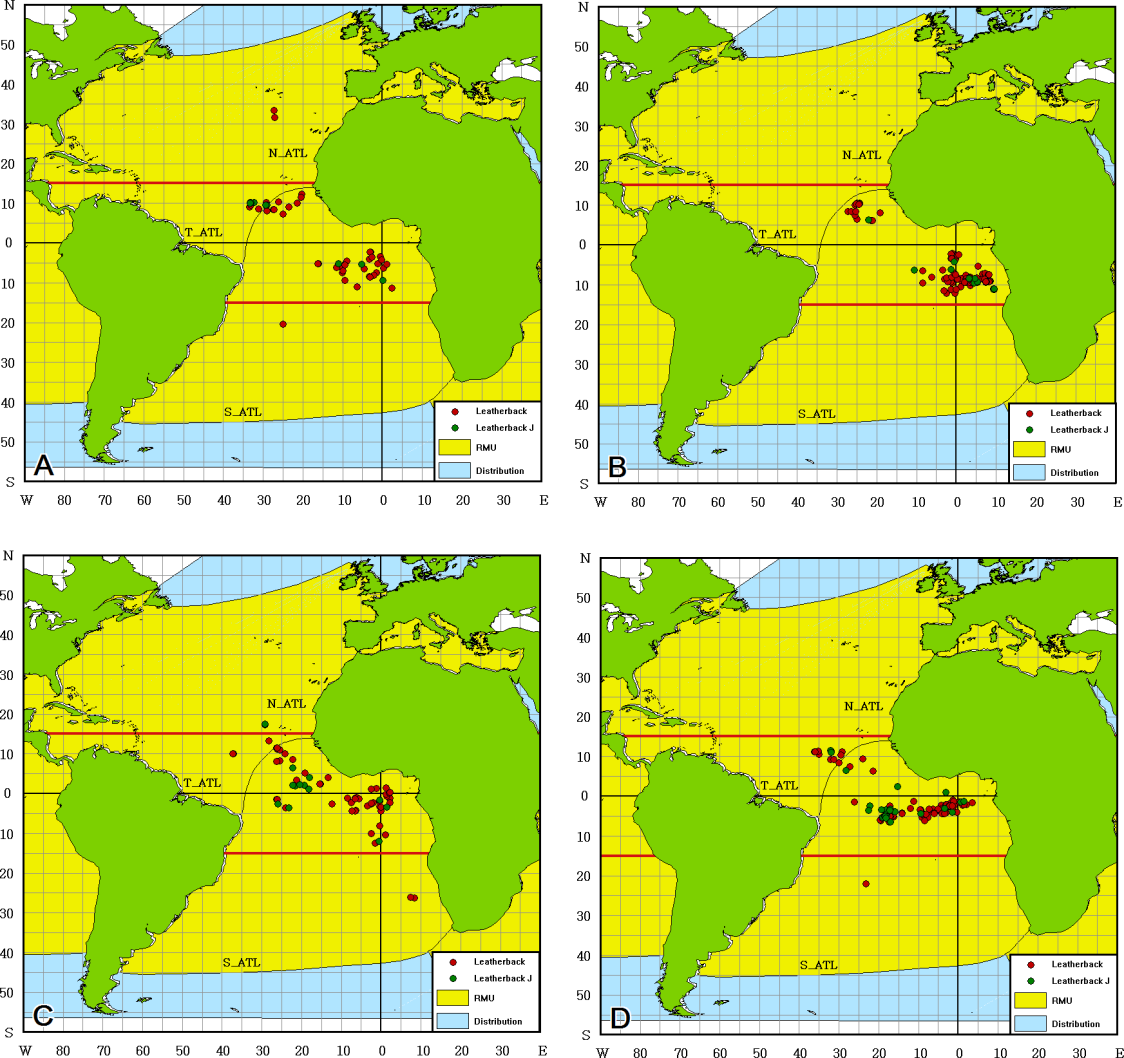
Distribution of leatherback turtle (*Dermochelys coriacea*) bycatch in the Atlantic Ocean by quarter. (A) January to March, (B) April to June, (C) July to September, (D) October to December. Juveniles are labeled by green points and adults are labeled by red points. The distribution Regional Management Unit (RMU, in yellow) is defined in accordance to [22]. The data was downloaded from the OBIS-SEAMAP/SWOT website (http://seamap.env.duke.edu/swot, [23–24]).

Most of the leatherback turtles (90.6%) were caught in TE_ATL in the Gulf of Guinea, especially in the second quarter (Fig 4). Most of the leatherbacks were caught in two areas, between 20–35° W and 5–15° N and 10°W-10°E and 0–15° S, from January to June. From July to December, the distribution was broader, from 35–0° E and 10° N– 5° S. More juvenile were distributed in the south of Equatorial and Gulf of Guinea (Fig 4). The BPUE values were high in T_ATL and ranged from 0.0017 to 0.0300 per thousand hooks per year (Table 2). The BPUE values were lower in N_ATL (0.0000–0.0104 per thousand hooks) and S_ATL (0.0000–0.0038 per thousand hooks).

#### Olive ridley bycatch

Olive ridleys were caught in waters ranging in temperature from 17.0–29.2°C with the majority being found in 26°C (Fig 2). Regarding the status onboard, 22.6% were alive, and 57.7% were dead (Table 3); most (81.7%) were hooked, and only a few (3.4%) were entangled (Table 4). In total, 27.4% were identified as female, and 16.8% were male (Table 5). The average length of the olive ridley turtles was 59.07 cm (49–121 cm range, SD: 8.59 cm, Fig 3B).

**Fig 5:**
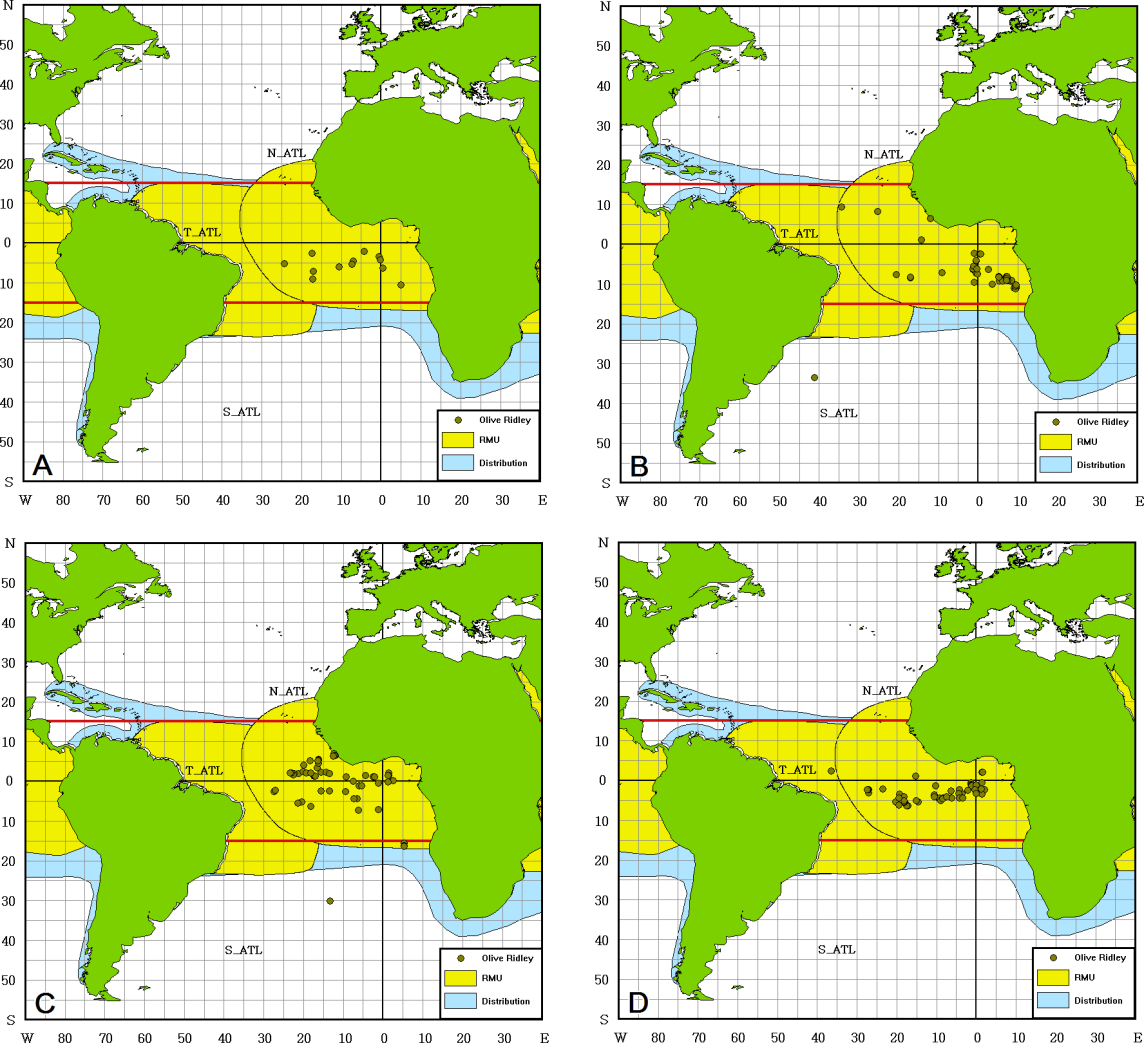
Distribution of olive ridley turtle (*Lepidochelys olivacea*) bycatch in the Atlantic Ocean by quarter. (A) January to March, (B) April to June, (C) July to September, (D) October to December. The distribution Regional Management Unit (RMU, in yellow) is defined in accordance to [22]. The data were downloaded from the OBIS-SEAMAP/SWOT website (http://seamap.env.duke.edu/swot, [23–24]).

Most of the olive ridleys (89.9%) were caught in tropical areas between 20° W and 5° E and 10° N and 10° S, and bycatch numbers were higher in the third and fourth quarters (Fig 5). The annual BPUE was highest in T_ATL and ranged from 0.0000 to 0.0232 per 1000 hooks. Since the olive ridleys are distributed in tropical areas only (Fig 5), there were no bycatch records in N_ATL and the BPUE in S_ATL was very low (0.0000–0.0032 per 1000 hooks) (Table 2).

#### Loggerhead bycatch

**Fig 6:**
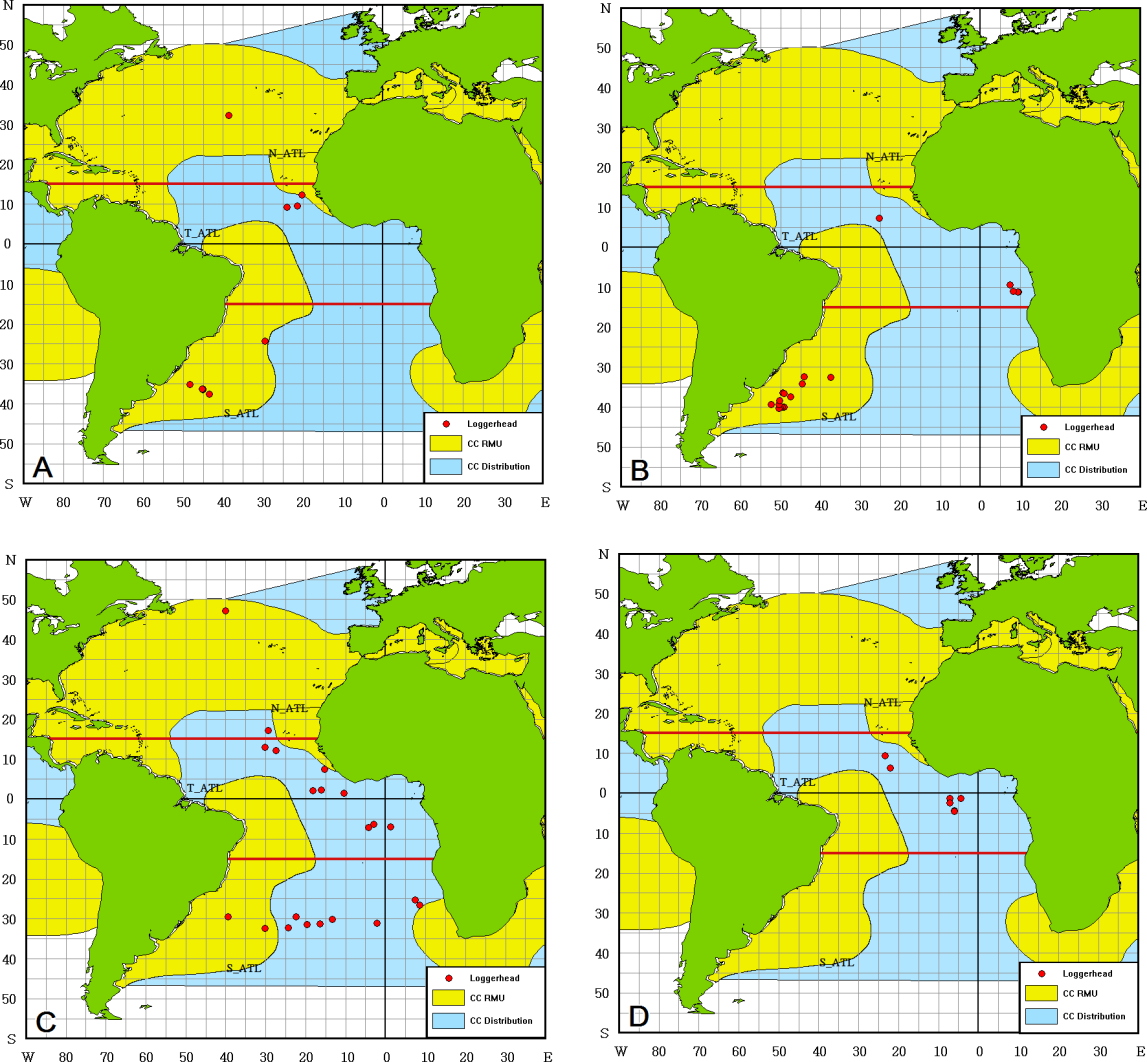
Distribution of loggerhead (*Caretta caretta*, *CC*) bycatch in the Atlantic Ocean by quarter. (A) January to March, (B) April to June, (C) July to September, (D) October to December. The distribution Regional Management Unit (RMU, in yellow area) is defined in accordance to [22]. The data were downloaded from the OBIS-SEAMAP/SWOT website (http://seamap.env.duke.edu/swot, [23–24]).

Loggerheads were distributed more to the south, and the SST ranged from 17.0°C—29.2°C with a higher percentage of turtles being found in 18°-20°C (Fig 2). Regarding onboard status, 55.2% were alive, and 34.3% were dead (Table 3); most (82.1%) were hooked, and only 7.5% were entangled (Table 4). Among the 28.4% whose genders were identified, 19.4% were female, and 9.0% were male (Table 5). The average recorded CCL was 58.64 cm (43–121 cm range; SD: 10.61; Fig 3C). Loggerheads were incidentally caught in all three areas (Fig 6). Annual BPUE values ranged from 0.000–0.0239 turtles per 1000 hooks which was highest in S_ATL in 2006 (Table 2).

### Bycatch of other turtles

**Fig 7:**
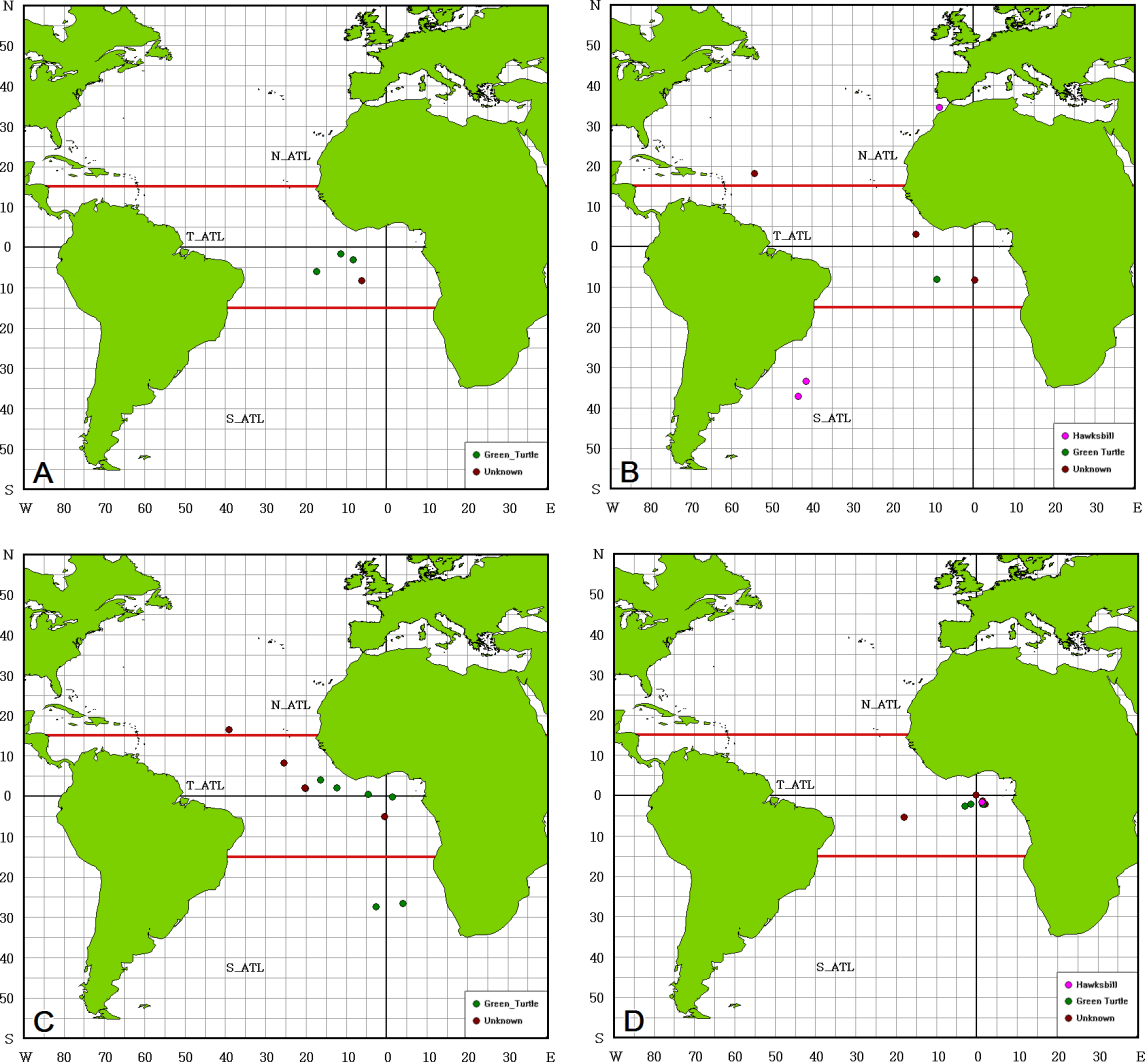
Distribution of bycatch of other turtles in the Atlantic Ocean by quarter. (A) January to March, (B) April to June, (C) July to September, (D) October to December.

Fourteen green turtles were caught, and they ranged from 46 cm to 64 cm in length (mean: 56.63 cm and SD: 5.88 cm). Most were distributed in T_ATL with only two in S_ATL (Fig 7). Fifty percent were released alive (Table 3); 78.6% were hooked (Table 4), and 21.4% were identified as female (Table 5). Six hawksbills were caught, and they ranged from 45 cm to 67 cm in length (mean: 57.60 cm and SD: 8.59 cm). All came onboard alive, and 66.7% were hooked (Tables 3 and 4). They were relatively equally distributed in T_ATL and S_ATL.

## Discussion

Much research has been performed in coastal areas to identify the status of sea turtle bycatch [11,14,16], but there is limited information about bycatch on the high seas. This study is the first to highlight the spatial-temporal distribution of the sea turtle bycatch of deep-set longline fleets on the high seas, and it fills an important data gap, especially that of the status of bycatch in the tropical high seas, the Gulf of Guinea, and the south Atlantic Ocean. In addition, the data provided additional valuable information about the distribution of loggerheads, which will inform the establishment of RMUs in the waters of the southern Atlantic Ocean.

The bias for estimation of bycatch number and mortality might be caused by many factors, including observer effect, unobserved mortality, underreporting of mortality, non-random allocation of sampling effort, logistical constraints, and inappropriate stratification [31, 37]. Babcock et al.[31] suggest that the observer coverage should be more than 20% for estimation mortality for target species and higher for non-target species. The limitation of this study is that the observer coverage rates were mostly lower than 20%, so it is not reliable to estimate the total mortality of sea turtles by species. Although the observed sets were low in N_ATL and S_ATL, especially before 2006, the variation of bycatch rates was high. On the other hand, the observed sets in the tropical areas were more than 12,000 sets during 2002 to 2013 in total, and the coverage rates in 2006 were 68.3%, which indicates that the bycatch rates in the tropical areas should be more reliable due to larger sample size compared to other fleets operating in the high seas.

In view of providing the best available data with a precautionary approach, this research showed the long—term bycatch rates in 12 years and annual variation of sea turtles bycatch number and mortality. Although higher observed sets might cause higher observation of bycatch cases, there is no correlation between number of observed efforts and bycatch rates (Table 2). The variation among years might be because of fishing areas, turtle abundance, etc. Bycatch is highly related to the location of the nesting grounds and migration routes of turtles [9], and previous bycatch research has shown variation among different areas. This research showed that the leatherback and olive ridley turtle bycatch rates were high in the tropical areas. This is compatible with previous studies because the tropical areas are major distribution areas for these two species.

In coastal areas, previous research has found weighted median BPUE values of 0.5954 turtles per thousand hooks in the northwest Atlantic region, 0.0367 turtles per thousand hooks in the northeast Atlantic region, 0.2240 turtles per thousand hooks in the southwest Atlantic region, and 0.274 turtles per thousand hooks in the Mediterranean region [11]. The turtle bycatch rates of the Taiwanese fleets were lower than most of these. In general, there are two possible reasons for low bycatch rates. First, the Taiwanese fishing grounds were mostly distributed on the high seas, where there are lower densities of sea turtles than in the coastal areas. Second, the operating depths were deeper than in the coastal or swordfish longline fisheries, and most turtles are distributed in shallower waters [38–40]. This phenomenon is supported by the higher numbers of leatherback bycatch as leatherbacks are distributed in deeper waters compared to other species.

The leatherback is the major bycatch species of the Taiwanese fleets, and most of the turtles were distributed in T_ATL (Fig 4); Japanese fleets have caught leatherback turtles in the Atlantic Ocean [41]. If estimated from the original catch and effort data, the nominal Japanese bycatch rate would be 0.0126 (183 leatherbacks for 14563 thousand hooks), which is similar to that of the Taiwanese fleet. The high bycatch areas for the Japanese fleets are also similar to that for Taiwan, the T_ATL, from October to March. These leatherbacks could have come from the northwest, southwest and southeast RMUs but not from southwest Indian RMU. Bycatch was limited to between 15°N and 15°S and was more concentrated in tropical areas. Eckert [36] showed the smaller leatherbacks (<100 cm) were only distributed in waters warmer than 26°C. The current study showed a similar result. Among 18 smaller leatherbacks, only 22% were caught in 23–25°C waters. The others were distributed in warmer waters (Figs 2 and 3). Fossette et al [42] analyzed leatherback satellite tracking data and fisheries data to identify possible bycatch areas, and Dodge et al[43] showed that leatherbacks aggregate in temperate waters during the summer and are more widely dispersed in the subtropics and tropics during the late fall, winter and early spring in the Northwest Atlantic Ocean. This research showed a similar trend for the East Atlantic Ocean; greater bycatch was observed in temperate waters in the summer and in tropical waters in the winter. This seasonal variation might be related to temperature, productivity, prey availability, and the necessity to spawn or nest in tropical regions. In addition, although Dodge et al [43] considered the offshore of Guinea Bissau to be low risk, this research did reveal bycatch in the waters. If BPUE is considered to be an abundance index, the high bycatch rate might be consistent with the high density of leatherbacks in the Atlantic Ocean. It is estimated that there are 28000 leatherbacks along the coast of West Africa [44], which is the highest number among the three Oceans, and the number of leatherbacks in the Atlantic Ocean is increasing due to certain conservation actions [45,46].

The olive ridley was considered to be the most abundant of all of the sea turtles in this study. Although the number of olive ridley turtles in the western Atlantic is higher than in the eastern Atlantic Ocean, the species accounted for most of the bycatch in the east (Fig 5) as those turtles might be migrating from their nesting grounds in central Africa [47,48]. Olive ridleys stay in waters less than 60 m deep [49]; thus, their bycatch rates might be lower than that of the leatherbacks.

Loggerheads are common bycatch of the United States fishing fleets in the north Atlantic Ocean and the Brazilian and Uruguayan fleets in the southwest Atlantic Ocean [50,51] The estimated number of bycatch mortalities from US fleets from 1990 to 2007 was 166900 and 26500 for loggerheads before and after regulation, respectively [52]. The fleets of Brazil, Uruguay, South Africa and Spain caught more loggerheads in the south Atlantic Ocean during this time [11]. In this study, it was shown that the distribution of bycatch areas in the central tropical and southern Atlantic Ocean (Fig 6); the bycatch was greater in the high seas during the non-breeding season (second and third quarter). These distribution information would be valuable for the identification of RMUs in the southern Atlantic Ocean, and it might be necessary to collect specimens for genetic analysis to confirm the origin of the nesting stocks.

[53] showed that the loggerheads distributed in US waters were found in a mean SST of 21.8 ± 3.0°C and a range of 12.8–30.4°C, and [54] showed that loggerheads were distributed at depths of 40–60 m and limited below 70 m. In this study, the important bycatch areas were the major fishing grounds of the albacore fleet, where the fishing depth is shallower than that of the bigeye tuna fleet. The loggerheads that were caught were mostly juveniles; only 18.0% were sub-adults and adults (>70 cm CCL). This is similar to the result for Chilean swordfish fisheries but higher than the bycatch rate in the artisanal fisheries of Peru [26], where gear selection (size of hooks) is possible reason.

The bycatch of other species was very limited and included only 14 green turtles, six hawksbills and 13 unknown species. Most of the bycatch green turtles were likely from the south-Central RMU as only two were caught in the SE_ATL (Fig 7). Green turtles usually inhabit coastal neritic habitats after completing their early juvenile stage. In the Atlantic Ocean, some countries allow direct take of turtles; Nicaragua, Colombia, Tonga, Sao Tome and Principe, and St. Vincent and the Grenadines are among the top ten countries with the highest legal take [55]. The number of turtles directly taken by countries ranges from more than 8000 to the hundreds of thousands, and green turtles comprise the highest proportion of the legal take. However, the direct catch of green, olive ridley, hawksbill, and loggerhead turtles has gradually been reduced since the 1990s; thus, fisheries bycatch might become the major threat [55]. Not only should the artisanal and coastal fleets be monitored because of their high bycatch rate, but considering the high effort by industrial fishing fleets, the status of bycatch should also be monitored on the high seas.

Although the bycatch rates of Taiwanese fleets were not higher than other coastal fleets, the survival rates of bycatch sea turtles were lower than other fleets because the Taiwanese fleets took more than 20 hours to complete one set. Furthermore, since some bycatch turtles were discarded before being placed onboard and some turtles might die after release, the real bycatch rates might be higher than the observed figures. In addition, the sea turtle bycatch was not completely evaluated in all related longline fleets. One should be cautious on the impacts due to Taiwan and other fishing countries. Each relevant fleet should share the responsibility.

Although closing certain areas to fishing is considered to be an effective conservation measure to reduce sea turtle bycatch [56], it would be difficult to implement on the high seas given the more expansive fishing grounds of industrial fleets. Gardner et al. [57] analyzed the sea turtle bycatch data and found the distribution of loggerheads and leatherbacks featured spatial-temporal patterns. The patterns, or hotspots, might be useful to the fishermen to avoid bycatch of turtles. In addition, Dynamic Ocean Management would be an option for sea turtle conservation [58, 59]. The TurtleWatch program has established a framework for sharing sea turtle distribution information in order to reduce bycatch by posting sea turtle information online every several days [58]. For example, the Taiwanese observer Office could collect real-time sea turtle bycatch information and post this information online to inform fishermen to avoid fishing at the hotspots. Furthermore, if a global turtle distribution data platform could be organized internationally, it would be helpful to provide more information to a variety of fleets from different countries. Other options include modifications of gear and fishing patterns; adjusting baits (finfish only) and using circular hooks have proved to be effective at reducing sea turtle bycatch in many longline fisheries [60–63]. However, because leatherbacks feed on gelatinous zooplankton, bait and hook modifications are less effective than for other species [64]. The higher percentage of entangled turtles confirms that bait is not the major reason for bycatch. The observer data showed that most turtles were caught by the first two hooks between floats; thus, it might be effective to reduce the use of these shallow hooks.

This study showed that bycatch rates might vary by year, area, quarter, and temperature. Hence, strengthening sea turtle data collection is an important task to improve the understanding of sea turtle bycatch and ecology. For example, regarding gender information, more than half of turtles could not be identified due to smaller size or larger size causing inaccessibility. Among those identified, females dominated. Since sea turtle gender is determination by temperature, future analysis of the sea turtle gender spatial-temporal distribution would be helpful for sea turtle ecology [65]. Bycatch method (hooked /entangle position), bait (squid or finfish), and survival status (healthy, injured, or coma, dead) data is valuable to evaluate possible measures for decreasing mortality. We suggest that these three parameters, at the very least, must be placed into the observer data collection system. In addition, low observer coverage and data deficiency in some areas might cause an underestimate of fishing mortality on sea turtles, especially in the years prior to 2006. Considering the bycatch rates will be impacted by quarter and areas, it is important to ensure a certain coverage rate in each stratum each year.

In conclusion, this research helped fill the information gap about bycatch on the high seas. The characteristics of these fleets are different from those of the coastal longline fishing vessels; they have a lower bycatch rate and higher mortality. Despite the lesser impact of Taiwanese deep-set longline fleets on turtles compared to other fleets, continued data collection and monitoring is necessary. Considering the broad distribution of the Taiwanese fleet across the whole Atlantic Ocean and the low coverage by observers, it would be helpful to use stratified sampling to direct observer deployment, such as increasing observer coverage in the north and south Atlantic Ocean.

## Supporting Information

S1 Appendix Results of multivariate analysis of variance for three bycatch sea turtles species. (DOCX)
