# Supplementary material for: Conservation Hotspots for the Turtles on the High Seas of the Atlantic Ocean
Source: PLoS One. 2015 Aug 12;10(8):e0133614. doi: 10.1371/journal.pone.0133614 (PMC4534298; doi:10.1371/journal.pone.0133614)
Supplement: S1 Appendix — (DOCX) [file pone.0133614.s001.docx]

S1 Appendix Results of multivariate analysis of variance for three bycatch sea turtles species

Table A ANOVA table for multivariate analysis of variance fitted to the bycatch rate of leatherback turtles in the Taiwanese tuna longline fisheries between 2002 and 2013.

| Source | DF | Sum of Squares | Mean Square | F Value | Pr > F |
| --- | --- | --- | --- | --- | --- |
| Model | 37 | 298.116 | 8.057 | 10.90 | <.0001 |
| Error | 17676 | 13063.399 | 0.739 |  |  |
| Corrected Total | 17713 | 13361.516 |  |  |  |

| Source | DF | Type III SS | Mean Square | F Value | Pr > F |
| --- | --- | --- | --- | --- | --- |
| Year | 11 | 161.347 | 14.668 | 19.85 | <.0001 |
| Quarter | 3 | 32.961 | 10.987 | 14.87 | <.0001 |
| Area | 2 | 13.482 | 6.741 | 9.12 | 0.0001 |
| SST | 21 | 8.078 | 0.384 | 0.52 | 0.9641 |

Table B ANOVA table for multivariate analysis of variance fitted to the bycatch rate of olive ridley turtle in the Taiwanese tuna longline fisheries between 2002 and 2013.

| Source | DF | Sum of Squares | Mean Square | F Value | Pr > F |
| --- | --- | --- | --- | --- | --- |
| Model | 37 | 109.760 | 2.966 | 6.35 | <.0001 |
| Error | 17676 | 8253.735 | 0.467 |  |  |
| Corrected Total | 17713 | 8363.495 |  |  |  |

| Source | DF | Type III SS | Mean Square | F Value | Pr > F |
| --- | --- | --- | --- | --- | --- |
| Year | 11 | 62.2948 | 5.6631 | 12.13 | <.0001 |
| Quarter | 3 | 17.6547 | 5.8849 | 12.60 | <.0001 |
| Area | 2 | 4.5259 | 2.2629 | 4.85 | 0.0079 |
| SST | 21 | 5.6929 | 0.2711 | 0.58 | 0.9342 |

Table C ANOVA table for multivariate analysis of variance fitted to the bycatch rate of loggerhead turtle in the Taiwanese tuna longline fisheries between 2002 and 2013.

| Source | DF | Sum of Squares | Mean Square | F Value | Pr > F |
| --- | --- | --- | --- | --- | --- |
| Model | 37 | 40.0549 | 1.0825 | 5.47 | <.0001 |
| Error | 17676 | 3499.9209 | 0.1980 |  |  |
| Corrected Total | 17713 | 3539.9759 |  |  |  |

| Source | DF | Type III SS | Mean Square | F Value | Pr > F |
| --- | --- | --- | --- | --- | --- |
| Year | 11 | 20.0669 | 1.8243 | 9.21 | <.0001 |
| Quarter | 3 | 0.3366 | 0.1122 | 0.57 | 0.6369 |
| Area | 2 | 0.6992 | 0.3496 | 1.77 | 0.1711 |
| SST | 21 | 16.7529 | 0.7977 | 4.03 | <.0001 |
